# Visual stimulus structure, visual system neural activity, and visual behavior in young human infants

**DOI:** 10.1371/journal.pone.0302852

**Published:** 2024-06-18

**Authors:** Marc H. Bornstein, Clay Mash, Martha E. Arterberry, Amir Gandjbakhche, Thien Nguyen, Gianluca Esposito

**Affiliations:** 1 Eunice Kennedy Shriver National Institute of Child Health and Human Development, National Institutes of Health, Bethesda, Maryland, United States of America; 2 Institute for Fiscal Studies, London, United Kingdom; 3 United Nations Children’s Fund, New York, New York, United States of America; 4 Colby College, Waterville, Maine, United States of America; 5 Department of Psychology and Cognitive Science, University of Trento, Trento, Italy; Federal University of Paraiba, BRAZIL

## Abstract

In visual perception and information processing, a cascade of associations is hypothesized to flow from the structure of the visual stimulus to neural activity along the retinogeniculostriate visual system to behavior and action. Do visual perception and information processing adhere to this cascade near the beginning of life? To date, this three-stage hypothetical cascade has not been comprehensively tested in infants. In two related experiments, we attempted to expose this cascade in 6-month-old infants. Specifically, we presented infants with two levels of visual stimulus intensity, we measured electrical activity at the infant cortex, and we assessed infants’ preferential looking behavior. Chromatic saturation provided a convenient stimulus dimension to test the cascade because greater saturation is known to excite increased activity in the primate visual system and is generally hypothesized to stimulate visual preference. Experiment 1 revealed that infants prefer (look longer) at the more saturated of two colors otherwise matched in hue and brightness. Experiment 2 showed increased aggregate neural cortical excitation in infants (and adults) to the more saturated of the same pair of colors. Thus, experiments 1 and 2 taken together confirm a cascade: Visual stimulation of relatively greater intensity evokes relatively greater levels of bioelectrical cortical activity which in turn is associated with relatively greater visual attention. As this cascade obtains near the beginning of life, it helps to account for early visual preferences and visual information processing.

## Introduction

Before they have much (if any) experience, infants selectively attend visually to different stimuli in the environment. *Why*?

Understanding patterns of infant attention and information processing is important because the world offers myriad sources and types of stimulation and information, and central challenges for human infants from the start of life are to monitor the environment by deploying their attention selectively, extracting critical information from the environment, and at the same time avoid being overwhelmed by a rich environment of constantly changing stimulation [1]. The complementary task for students of early human sensory, perceptual, and cognitive development is to understand how young infants overcome those challenges. One early (but infrequently and not yet comprehensively tested) hypothesis suggests a cascade: infant visual attention to selective visual stimulation is mediated by excitation of neural tissue along the visual pathway [2,3]. Individual neurons as well as cell assemblies are known to respond selectively to different types of visual stimulation–orientation, direction, and hue, for example. Classic neurophysiology attests that, when the appropriate stimulus or “trigger feature” for a given neuron enters its receptive field, the rate of the neuron’s electrical discharge increases [4]. Stimuli that deviate from the neuron’s preferred trigger feature produce lower rates of firing or even inhibit neuronal excitation from its spontaneous level. This stimulus-response “dose” relation between specific stimulation and central nervous system activity is well established [5]. Notably, Hubel and Wiesel began their pathbreaking neurophysiological research with the straightforward assumption that turning on or turning off diffuse light in front of a cat’s eyes ought to affect the activity of cells in the cat’s brain. As Hubel recounted in his Nobel laureate lecture, to their surprise, however, Hubel and Wiesel found that neurons in the cat visual cortex were not excited by the mere presence or absence of light [6]. Instead, specific cells at specific cortical sites were excited by specific visual contours rotated to specific orientations moving in specific directions. Such specificities of stimuli, topography, velocity, and the like have since proven to be the rule in nervous system function [7,8]. On this classic principle, feature-detecting neurons code specific dimensions of environmental stimulation and mediate eventual behavior in the fashion of a cascade.

Although precise relations among environmental stimulation, neural coding, and perception and information processing are complex and still poorly understood, it has been hypothesized in developmental science, but not adequately nor comprehensively tested to date, that how much visual attention that a stimulus structure elicits from an infant may be governed by how much neural activity that stimulus structure excites. Here, in two related experiments we tested that proposition directly by examining both visual preference and cortical electroencephalographic (EEG) responses to variation in specific visual stimuli in human infants and (for comparison) adults. For reasons explained below, the visual structure we manipulated was chromatic saturation, the index of visual system neural activity at the cortex was event related spectral perturbation, and the measure of infant visual behavior was looking time. So, null hypotheses would be that stimulus saturation level has no effect on infant brain electrical activity and saturation level has no effect on infant looking behavior.

A brief review of the history of infant testing that anticipated this cascade proposition led us to these specific hypotheses. As long ago as the late 1950s, Berlyne observed that infants prefer to look at patterns with more contour, and he presciently speculated, based on then current physiological understanding, that patterns with more contour excite fibers along the visual system more [9]. This ‘‘high stimulating power,” Berlyne argued, accounts for the “high eye-drawing power of patterns.” Kessen and colleagues later recorded neonates’ eye-movement scanning and documented a preference for newborns crossing vertical edges; in turn, they speculated that that preference reflected greater neural stimulation provoked by vertical contours [10]. Subsequently, Karmel, Hoffman, and Fegy recorded visually evoked potentials (VEPs) from the cortex of infants who looked at checkerboards of different patterns [11]. The longest infant total looking times were to patterns that evoked maximum amplitude VEPs (see also [12]). Soon after, Karmel and Maisel confirmed that infants look more or less at patterns with more or less amounts of contour, and they hypothesized that patterns with more contour optimally stimulated cells in the visual cortex tuned to contour detection [13]. With such data in hand, Haith advanced the principle that cortical firing rate guides infant vision, and he generated a series of related “rules” to account for infant visual search [3]. However suggestive, these studies together do not provide integrated support for a cascade from visual stimulus structure to neuronal activity along the retinogeniculostriate visual system to visual preferential looking in infants. The main purpose of the experiments presented here was to provide that more integrated and direct experimental test of the cascade.

Color is a basic domain of vision and a pervasive aspect of human visual experience. The subjective organization of color perception is tridimensional, encompassing hue, brightness, and saturation [14]; hue is most closely associated with dominant wavelength, brightness with luminance, and saturation with purity as physical variables [15]. Color is an esthetically impressive, perceptually appealing, and cognitively informative aspect ofthe visual world [14,16]. Colors possess influential affective qualities as they moderate mood [14]; they elicit and maintain visual attention, as they clarify and accentuate features of the visual environment [17]; and they aid detection, identification, encoding, learning, and communicating about visual attributes and objects in the world [18–21].

Chromatic saturation provides a functional stimulus dimension to test the cascade hypothesis. Saturation is the subjective density or vividness of color in a mixture with white and is a continuous variable. Saturation (*aka* chroma) corresponds to the physical property of purity. In the present experiments, we used levels of chromatic saturation to investigate neural responses and behavioral preferences for two reasons. First, neonates [22], 2-month-olds [23], and 4-month-olds [2] reliably discriminate among levels of chromatic saturation and discriminate saturated colors from white. Relevantly, the developmental literature indicates that young infants also pay more attention to more saturated colors than to less saturated ones [2,23,24]. When infants as young as 4 months of age are shown two otherwise equivalent visual stimuli, one of which is a saturated color and the other is either white, neutral gray, or less saturated, viewed against dark or achromatic surrounds, infants regularly look at (prefer) the more saturated color of the two [2,23,25–27]. Indeed, infants’ looking behavior obeys what has been called a *Maximum Saturation Rule—*that is, along the purity dimension from white to a chromatic saturation maximum, infants’ visual preference minimum lies at the white end of the dimension and their visual preference (looking) increases with increasing stimulus saturation [26,28–30].

One experiment assessed infants’ preferences among six chromatic stimuli versus white, all tested at physical isoluminance and adult-judgement isobrightness. Infants preferred all isoluminant chromatic stimuli to white and preferred all chromatic stimuli judged by adults to be equivalent to white in brightness [31]. The second reason we chose chromatic saturation as the stimulus test dimension is because the neurophysiological literature indicates that more saturated colors (a) stimulate activity in neurons in the visual system that are sensitive to color and (b) increase amplitude of the visually evoked cortical potential in human newborns. With respect to visual system activity, De Valois and Marrocco [32] determined that the frequency of discharge of color-sensitive cells in the lateral geniculate nucleus [33] of the primate *Macaca irus* increases monotonically with the saturation purity of visual chromatic stimulation. Cells sensitive to long wavelengths (“red”) fired to white light around the rate of their spontaneous discharge, but they fired to increasingly monochromatic long-wave (λ = 640 nm) light with increasing frequency. With respect to newborn cortical electrical activity, purity of saturation influences the amplitude of the VEP. Lodge, Armington, Barnet, Shanks, and Newcomb [34] studied the neonatal VEPs to white and long-wave (λ > 580 nm) light. When stimuli were equated for brightness, more purely saturated “orange” produced visually evoked potentials in newborns at an amplitude three times greater than to white.

On the basis of these kinds of data, we hypothesized, first, that infants would preferably attend to (look at) otherwise equivalent more saturated than less saturated chromatic visual stimuli and, second, that infants would show greater cortical neural electrical activity to otherwise equivalent more saturated than less saturated chromatic visual stimuli. To test the first hypothesis, in Experiment 1 infant visual looking times (attention) to less and more saturated versions of the same chromatic visual stimuli were assessed where wavelength and brightness of those stimuli were held constant. To test the second hypothesis, in Experiment 2 infants’ and adults’ cortical EEG activity was monitored while they looked at the same visual stimuli of less and more chromatic saturation.

## General methods

### Participants

Infants in the two experiments were born at term and 6 months of age and healthy at the time of testing. We recruited infants aged 6 months because research confirms that by this age infants have trichromatic color vision [35–39]. Moreover, infants in this age range have the abilities to control their attention [40], and the design of these experiments implemented an infant-controlled visual perception procedure. Infants were recruited through the use of purchased mailing lists of newborns in a suburban metropolitan area and represented families of middle to upper socioeconomic status. Sample sizes in these experiments were determined by reference to previous related research and are consistent with a predominance of infant visual attention studies [41,42]; data collection ceased when planned sample sizes were reached; and attrition rates were comparable to other published looking time and EEG studies [41,43]. Adults were recruited from Institutional staff. Each participant in these studies (infants and adults alike) were first-time participants; these were the only such studies in which they participated; and all participants were naïve as to the hypotheses of the experiments. Participants were tested in accordance with ethical principles of the Declaration of Helsinki, and the research Clinical Protocol 04-CH-0250 was approved (11/16/2011) by the Institutional Review Board of the *Eunice Kennedy Shriver* National Institute of Child Health and Human Development. The recruitment period of the study was 5/1/2010–11/14/2013. Infant participants’ parents and adult participants were informed about study procedures before participating. Consent for participation by infants was obtained by parents’ signature, and for adults by each participant’s signature.

#### Stimuli

**Fig 1:**
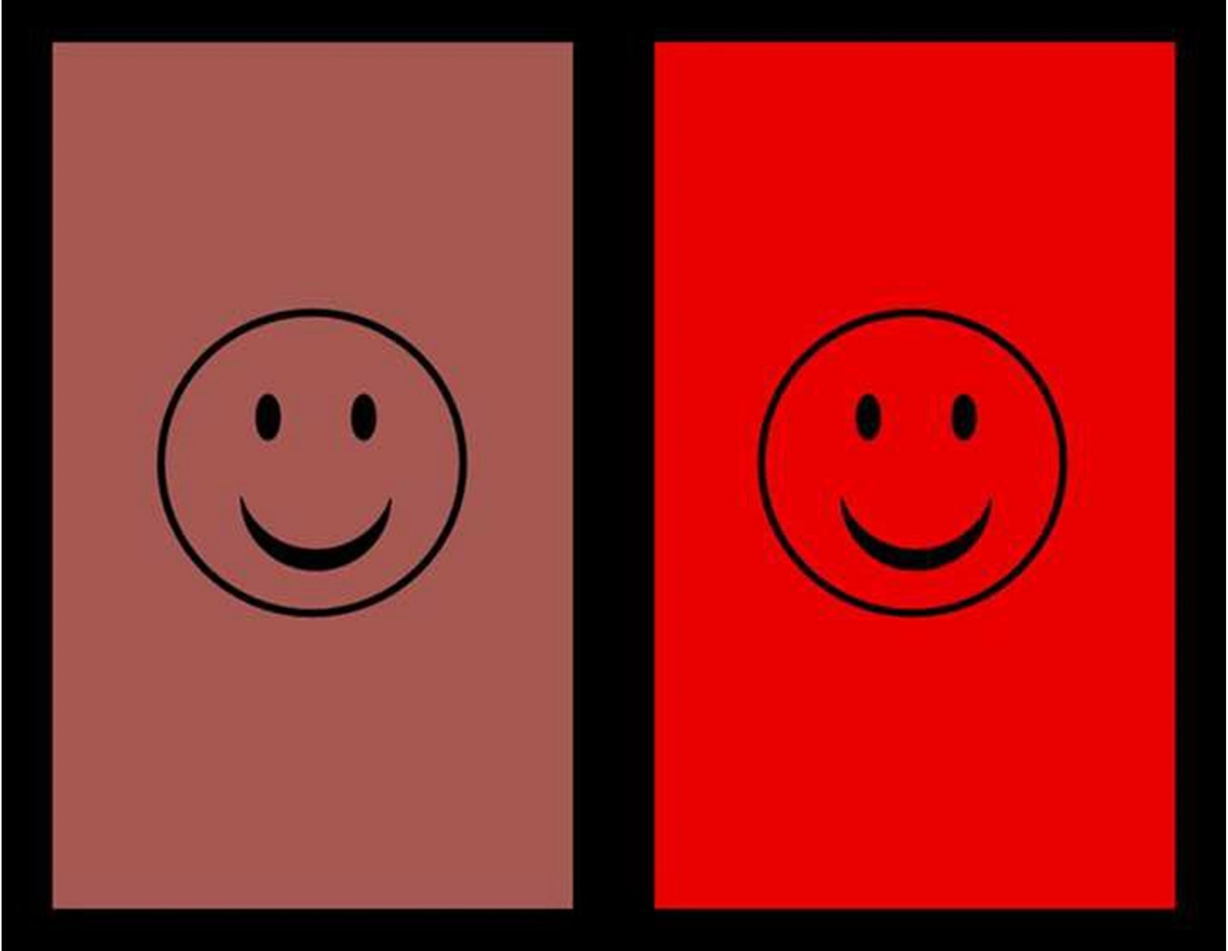
Stimulus exemplars from Experiment 1A.

The same chromatic stimuli were used in Experiments 1 and 2. Table 1 shows their CIE Yxy coordinates. Two 17.4° by 22.6° chromatic fields were generated by a computer program (E-prime; Psychology Software Tools, Pittsburgh, PA) with the same 630 nm dominant wavelength (“red”) and equivalent brightness and presented on a computer monitor (Dell UltraScan P1110 21”; Dell Technologies, Round Rock, TX) against a black background (as has been done previously; [37,44]). Each stimulus field contained a simple circular schematic face in black outline that was 8.7° in diameter and centered within the field (Fig 1). The faces were intended to maintain infants’ engagement with the task without biasing attention, and they were sized to be salient in relation to infants’ developing acuity while still fitting parafoveal limits when centrally fixated. One field was more saturated and the other field less saturated. Because stimulus brightness can vary independent of saturation [45] and affect infant attention, brightness (as well as wavelength—hue) was held constant with the use of isoluminant chromatic stimuli. This selection enables interpretation of any observed behavioral and psychophysiological differences as occurring in relation to saturation uniquely. Infant and adult isoluminance values are similar [46], and infant and adult spectral sensitivity for video generated stimuli are also similar. Moreover, the amplitudes and latencies of the major components of chromatic onset responses represent robust to large (even intentional) luminance mismatches [47,48].

**Table 1:** CIE Yxy coordinates for chromatic stimuli used in Experiments 1 and 2.

Chromatic Stimulus	Y	x	y
**“Red” more** **saturated**	17.66	0.64	0.33
**“Red” less** **saturated**	15.71	0.45	0.34
**“Green” more** **saturated**	37.26	0.30	0.60
**“Green” less saturated**	36.08	0.31	0.48

### Specific methods and results

#### Experiment 1A

Experiment 1A assessed infants’ spontaneous looking preferences between the more and less saturated stimuli when presented side by side. Ruff [49] presented 4.5-month-olds with paired high-contrast geometric stimuli, and she argued that infants’ gaze shifts indexed active efforts to process and compare paired stimuli. Infants may be more motivated (e.g., by attentional salience) to make eye movements towards chromatic stimuli with greater saturation than ones with lesser saturation [50].

#### Participants

Fifteen 6-month-old infants (*M* age = 24.5 w, *SD* = 1.2; 11 females) participated. Data from an additional 4 infants were not included due to inattentiveness during the procedure.

#### Stimuli and procedures

The more saturated and less saturated fields were presented simultaneously for trials that lasted 10 s each and alternated the left versus right location of the two saturated stimuli. We implemented an infant-controlled procedure, wherein each 10-s trial began when the infant was judged to be looking centrally at the stimulus screen (where the visual stimulus would appear). Intervals between trials therefore varied somewhat, averaging approximately 4 s.

Infants sat alone in an infant seat in a dimly lit room in front of the computer monitor at a viewing distance of 60 cm. An Applied Science Laboratories (ASL; Bedford, MA) Model 504 infant eye tracking system captured infants’ fixations on each stimulus image. The system used infrared corneal reflection to record fixation coordinates on the stimulus plane continuously at 60 Hz. An ASL optical face tracker corrected angles on the eye camera relative to spontaneous movements of the head that exceed optical-tracking frame limits. GazeTracker (EyeResponse Technologies, Charlottesville, VA) software, running on a second microprocessor, synchronized eye movement recordings with stimulus presentations. The eye camera was located beneath the monitor in the same depth plane as the monitor screen and recorded infant looking.

#### Results and discussion

**Fig 2:**
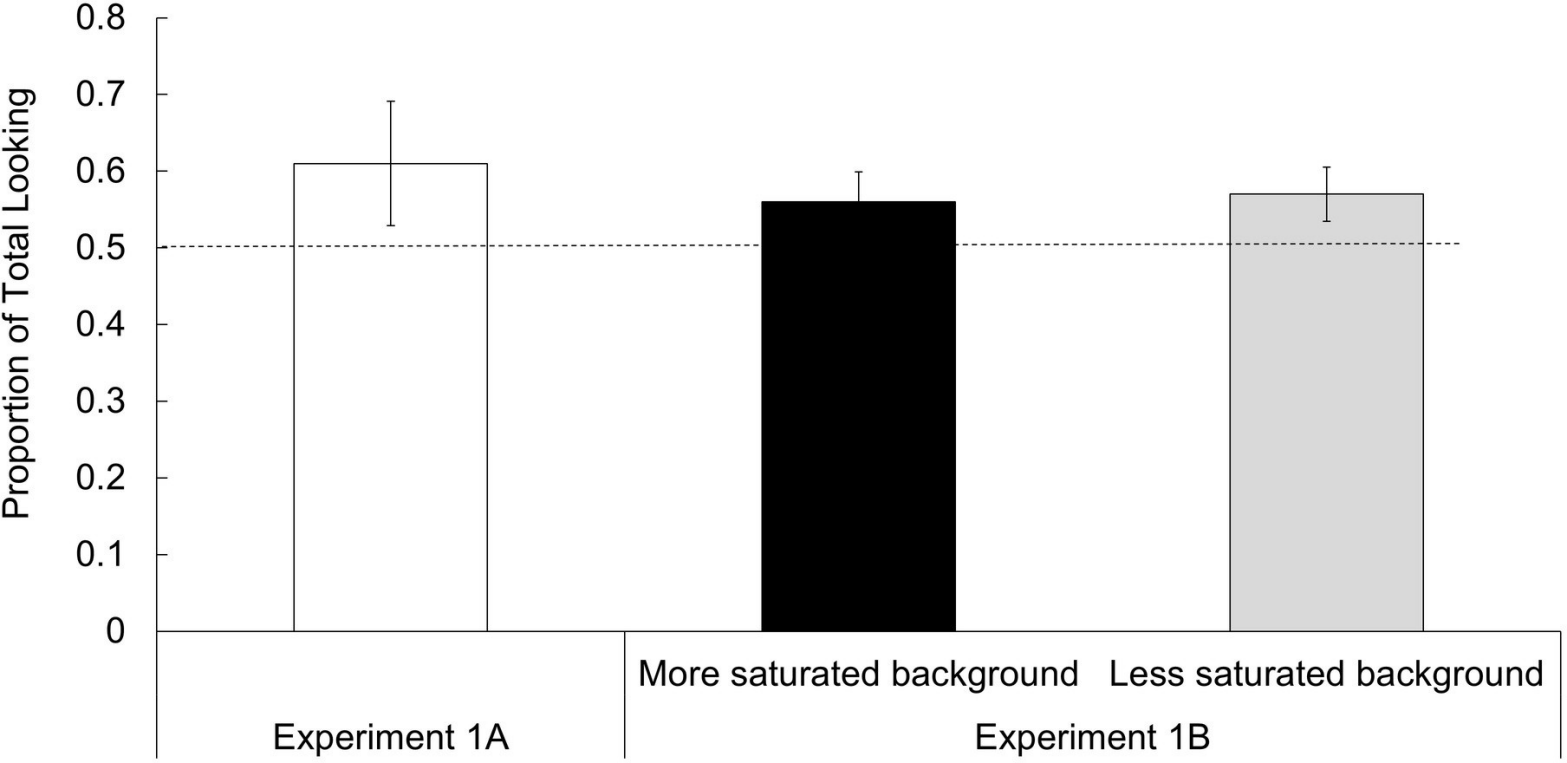
Mean (95% CI) proportions of total looking time directed to more saturated stimuli; the dashed line at 0.5 is chance.

For each trial, infant visual fixations (dwell times) were plotted directly on the stimulus fields (off-display fixations were disregarded). Following conventional practices [51,52], the durations of individual infant fixations to different saturation levels (inclusive of field edges) were totaled, and preference scores were determined by dividing the total duration for the saturated fields only by the total of all fixations. Using a one-sample *t*- test, these preference scores were then compared to chance (0.50; with 15 participants and estimates from previous research, power was estimated at 0.75). As hypothesized, infants preferentially fixated the more saturated stimulus displays over the less saturated stimulus displays, *M* = 0.61, *SD* = 0.16, *t*(14) = 2.70, *p* = .017, *g* = 1.41 [53] (see Fig 2).

Infants provided behavioral evidence of visually preferring the more saturated to the less saturated of otherwise equivalent (hue- and brightness- matched) chromatic stimuli. The results of Experiment 1A replicate Bornstein [2], who showed 20 4-month-olds six colors, three hues (two blues, greens, and reds with the same brightness) each at a low and high chromatic saturation singly for 15-s durations in different counterbalanced random orders. Infants in that study also looked more on average at the more saturated versions of hues than they looked at the less saturated versions of the same hues (see also [24]).

#### Experiment 1B

Experiment 1B assessed the influence of variation in foreground- background color saturation on infant spontaneous looking preferences. Because the relative saturation of background coloration can interact with infants’ spontaneous visual preferences [26], additional examination of infant preference for a more over a less saturated version of the same hue and brightness was warranted. Pereverzeva and Teller presented infants with chromatic stimuli of varying saturation levels against both white and chromatic backgrounds. Infants looked longer at saturated stimuli in the white-background condition, and the researchers concluded that infant preference was due to maximal difference in purity. Additional investigation in Experiment 1B was also motivated by the related idea that the visual system may be sensitive to relative signals more than absolute stimulus attributes.

#### Participants

Twenty-five 6-month-old infants (*M* age = 25.8 w, *SD* = 2.4; 8 females) participated in Experiment 1B. (Data from an additional 6 infants were not included due to inattentiveness.) A larger number of infants was recruited in Experiment 1B than in Experiment 1A because an additional critical test of effects was needed.

#### Stimuli and procedures

Experiment 1B differed from Experiment 1A only in the background used in stimulus presentations. The same paired “red” color fields from Experiment 1A were presented in Experiment 1B, this time against “green” (instead of black) backgrounds, with two different saturation levels of “green” used (see Table 1). The more saturated and less saturated “green” background levels were counterbalanced with the left/right panel positions of the two “red” field saturation levels across trials. All other aspects of the task were identical to those of Experiment 1A.

#### Results and discussion

Infants again preferentially fixated the more saturated red fields of the stimulus displays over the less saturated versions across both saturation levels of the backgrounds (*M* = 0.56, *SD* = 0.10, *t*(24) = 3.34, *p* = .003, *g* = 0.60, for stimuli with less saturated green backgrounds, and *M* = 0.57, *SD* = 0.09, *t*(24) = 3.55, *p* = .002, *g* = 0.74, for stimuli with more saturated green backgrounds). No difference was observed between background saturation levels. Under the conditions examined here, the saturation level of background coloration did not affect infants’ spontaneous preference for more saturated fields over less saturated ones. Thus, infant visual preference for relatively greater saturation is also robust to variation in chromatic background hue and saturation characteristics. Across both experiments, we used novel stimuli, controlled relevant stimulus characteristics, and presented stimuli only after infants were attending to the stimulus monitor. Thus, factors such as state and familiarity cannot account for the pattern of findings.

#### Experiment 2

Experiment 2 examined cortical activity in infants and adults to relatively less and more saturated chromatic stimuli of the same hue and brightness. Cortical responses to chromatic stimuli are robust, and electrical activity at the cortex in response to chromatic stimulation has become a sensitive and objective measure of neural development in infancy [54]. Experiment 2 used a task-based EEG paradigm in which EEG was measured to a specific set of stimuli with the aim to reveal real-time brain activity associated with looking at those stimuli.

#### Participants

Ten 6-month-old infants (*M* age = 26.7 w, *SD* = 1.1, 5 females) and ten adults (*M* age = 22.41 y, *SD* = 1.20, 7 females) participated. Data from an additional 16 infants were not included (8 due to fussiness or inattentiveness, 6 due to artifacts in the EEG data, and 2 due to equipment failure). This sample size is consistent with a predominance of infant visual attention studies [42].

#### Stimuli and procedures

**Fig 3:**
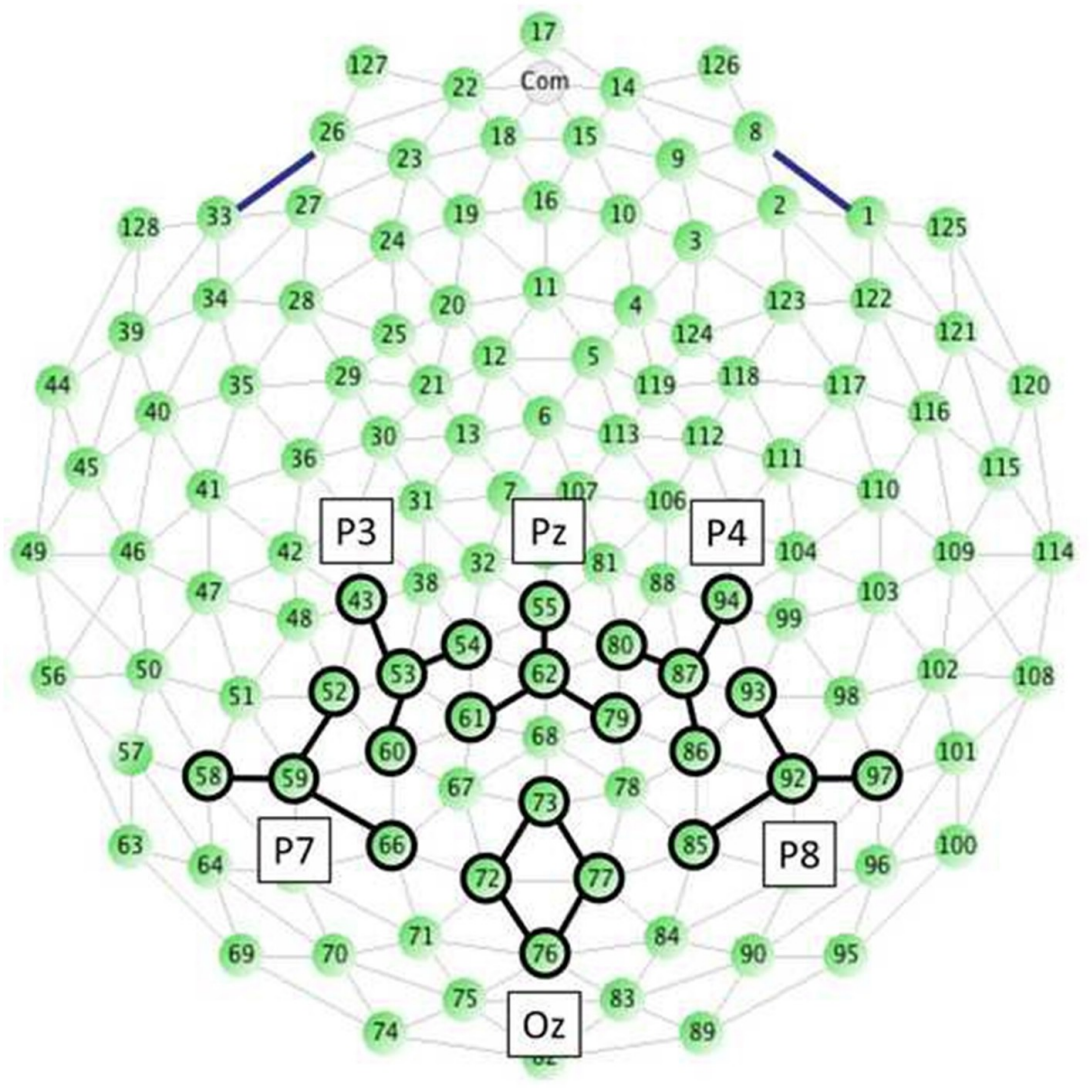
Recording site clusters used with infants.

Thirty trials of each of the two saturation levels were presented singly in random order for a total of 60 trials. On each trial, once infants were judged to be looking at the center of the stimulus monitor, a 300-ms baseline period preceded stimulus presentation; the stimulus appeared for 500 ms; then a variable duration 1800- to 2200-ms intertrial interval followed during which the presentation screen was black. The EGI 128-channel EEG system (Magstim EGI; Eugene, OR) was used to record cortical EEG. The international 10–10 system sites P3, Pz, P4, P7, Oz, and P8 were clustered and selected for analysis (Fig 3). EEG artifacts were detected and removed using the Automatic EEG Artifact Detection Based on Joint Use of Spatial and Temporal Features (ADJUST) [55,56] running in Matlab v8.0 (MathWorks; Chevy Chase, MD). Components corresponding to four classes of source artifact were removed: eye blink, vertical eye movement, horizontal eye movement, and generic discontinuity.

#### Results and discussion

Brain activity giving rise to the perceptual experience of color likely occurs at higher-level cortical areas upstream from the pre-cortical retina and lateral geniculate nucleus [57]. We therefore examined electroencephalographic cortical activity using event related spectral perturbation (ERSP), an index of changes in the spectral power of EEG frequency bands as a function of time following the onset of an experimental event [58–60]. The ESRP is a measure of mean amplitude changes in EEG frequency to an experimental event across time [61], and ESRP is especially useful because it allows extraction of meaningful EEG signals from few trials [62]. Independent component analysis (ICA) is used to identify EEG rhythms to data from a single trial during a time period inclusive of the time window of components. It finds spatiotemporal patterns of consistent frequency content in participants’ scalp topography. ERSP is calculated by segmenting the EEG signal into brief overlapping data windows, and a moving average of the amplitude spectra of the windows is calculated and divided by mean baseline spectra preceding the event. Spectral decomposition of signals from each channel proceeded with a Morlet wavelet transform [61]. A 100- ms window and wavelet cycles varying linearly from 2 to 9 were used to recover frequencies from 4 to 60 Hz over 57 equal-interval bins. The ERSP was used to examine time resolution of the spectral power evoked by stimuli assuming that attention increases with increased activation [59]. Among infants the relation between EEG response and attention depends on task demands and age but is well documented [63].

To identify predominant response timing, grand average time- frequency ERSP response maps were examined over trials, participants, stimulus saturations, and channels for each channel cluster. Infants’ initial responses appeared around 80 ms in the beta and lower gamma bands (23–43 Hz) and waned by 210 ms. Adult responses appeared around 30 ms in a similar range of frequencies (13–53 Hz) and extending through 235 ms.

Beta waves are prominent during intense mental activity, focus, active thinking, and alert concentration; gamma waves are prominent during higher-order cognitive processing and are thought to reflect the binding of different populations of neurons into networks for the purpose of carrying out a certain cognitive or motor functions [64,65].

**Fig 4:**
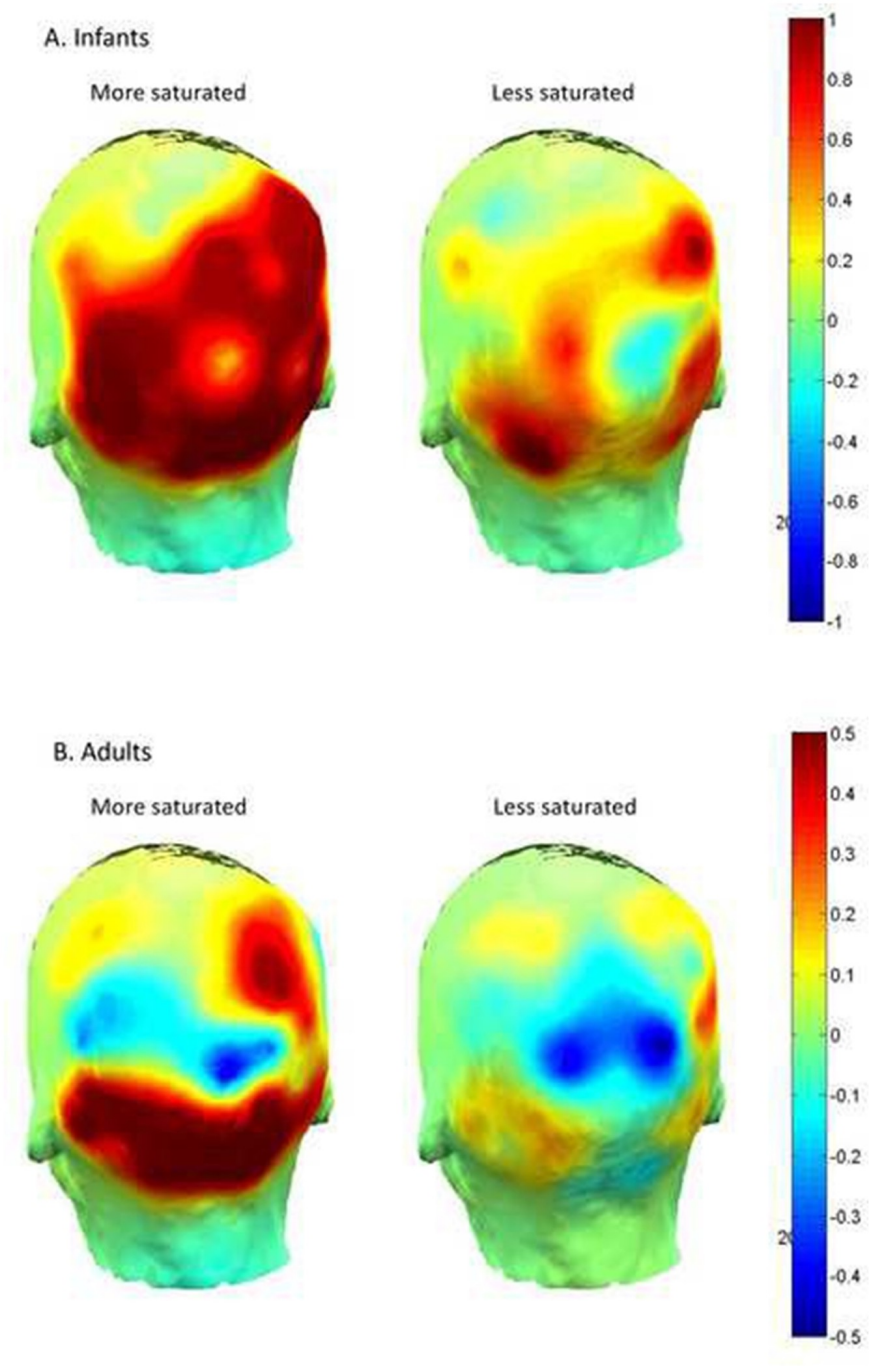
Topographic head maps of averaged ERSP responses across posterior sensors in A. Infants and B. adults.

**Fig 5:**
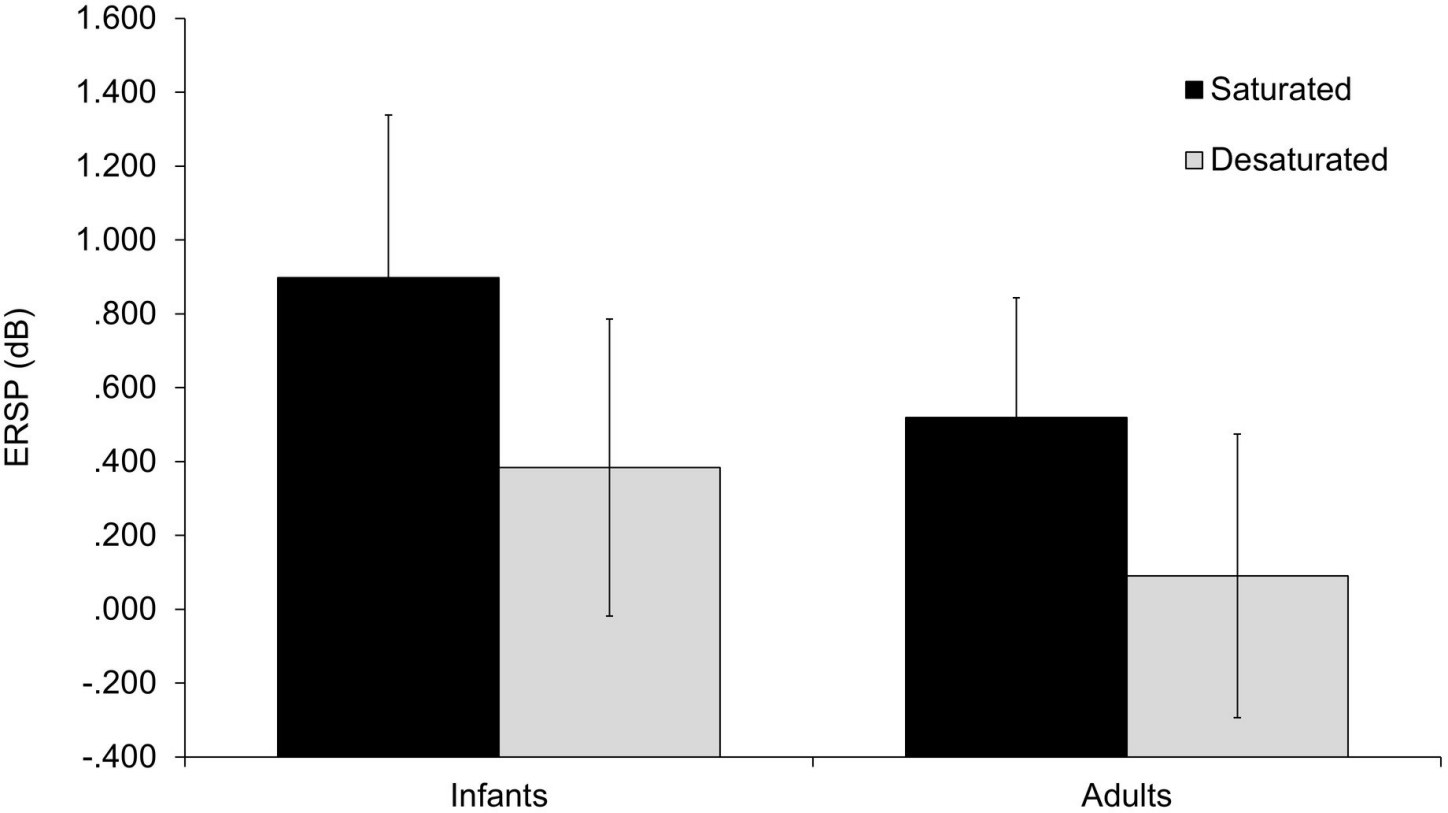
Mean (95% CI) spectral perturbations of responses to stimuli by age group and saturation level.

Fig 4 shows the topography of ERSP responses averaged over these selected time windows and frequency ranges by stimulus saturation level and age group. ERSP values were examined in a linear mixed models analysis including saturation level and recording site as repeated fixed effects, age group as a between-subjects fixed effect, 2- and 3-way fixed-effect interactions, and participants as a random effect (power to detect main effects based on previous research was estimated at 0.75). A significant effect of saturation level emerged, *F*(1,21.63) = 6.60, *p* = .018, ω_p_^2^ = 0.19, with the greater ERSP in response to the more saturated (*M* = .59, *SD* = .81) than less saturated chromatic stimulus (*M* = .15, *SD* = .79; see Fig 5). No other effects were significant.

## General discussion

Infants look longer at some features of their visual environment than at others, an observation that is historically well-grounded in the infancy studies literature [1,10,66–68]. Chromatic saturation is one such stimulus feature of the environment that draws infant attention. The reasons for such taxes have generated speculation. In these studies, infants and adults viewed otherwise equivalent stimuli of two contrasting levels of chromatic saturation, and their looking times and brain responses differed systematically between the two levels of chromatic saturation in both age groups.

In the first experiment, the preferential looking time behavior of 6- month-old infants was assessed. Infants displayed a preference for the more saturated over the less saturated chromatic stimulus as measured by the fraction of their looking time. The two stimuli were otherwise equivalent and presented against a neutral black background. Infants’ preference was also maintained against green backgrounds of low and high colorimetric purity indicating that background contrast was not the differentiating factor that influenced infant looking (attention).

In the second experiment, cortical activity was measured in both infants and adults in response to the same more and less saturated chromatic stimuli used in the first experiment. Neural signals were analyzed at a series of electrode positions across the scalp in the frequency domain in a specified time window. The more saturated chromatic stimuli generated greater cortical activity than the less saturated stimuli in both infants and adults. Qualitatively, adult response differences appear to be more localized to posterior parietal locations than those of infants, although the location effect was not significant in the analysis.

What accounts for the variation in infant attention to different levels of chromatic saturation? Explanations that include learning experience and reinforcement history are feasible as social interactions and experiences influence the development of sustained attention [69,70]. However, young infants in the present experiments likely enjoyed little if any learning experience and no reinforcement history with respect to chromatic saturation. Taken together, data from the present experiments rather support the hypotheses that how much visual attention infants pay to a visual stimulus reflects in a regular way how much neural activity in the visual system that stimulus excites [2]. Specifically, the two hypotheses advanced here were both supported. Other EEG and fNIRS studies reinforce the conclusion that infant preferential looking is correlated with responses of neural ensembles that encode given stimulus types and attention networks [71–74].

These experiments reveal infants’ relative visual preferences for more over less saturated versions of otherwise equivalent chromatic stimuli. These data also speak to two related issues in infancy and developmental science. First, these preference and neural data indicate that infants as young as 6 months of age discriminate levels of saturation of matched brightnesses within the same hue. Only one previous study has reported that infants discriminate chromatic saturation. There, 4-month-olds discriminated chromatic stimuli of moderate excitation purity (30 to 50%) from chromatic stimuli of lower purity (5–11%) [2]. Second, the replication documented here contributes to counteracting developmental science’s replicability problem [75,76].

As with all brain-behavior experiments, explanations of behavioral events in terms of neural events are accompanied by important limitations. For example, interpretations of attention often appeal to two key assumptions: One is that single-neuron or aggregate-neuronal data accurately reflect central nervous system information processing, and the other is that central nervous system activity relates to manifest behavior [7]. Both assumptions raise questions and problems. Whether and how activity of single neurons in the visual system relate to gross electrical activity at the cortex are not well worked out. VEPs might reflect numbers of active neurons or a novel ensemble of overall neuronal activity; these are two among many possibilities. Population coding could also involve different patterns of neutral activity, which might not translate into increased total neural activity over the population [77–80]. Moreover, single neurons, larger groups of neurons, or neural patterns of activity may be stimulated by different segments of a stimulus range. The connection between the intensity of a stimulus and the focus of visual attention may not be determinate (even when the amplitude of a VEP correlates with stimulus intensity) [81], and many variables such as expectancy and affect drive the shape of the VEP. All these aspects aside, attention as a psychological variable notoriously challenges definition [82,83]. Moreover, some nervous activity is uncorrelated with the magnitude of stimulation [84], and VEP amplitude is only a statistical summary of synchronous neural activity [13,85,86].

Relatedly, this study is concerned with visual preference, but visual preference and visual attention are not necessarily congruent. That is, attention is a likely antecedent of preference in the sense that an observer must attend to a stimulus before any expression of preference, but whether preference is a valid measure of attention is less clear as it is possible to look at something and not “attend” to it in any deeper information processing sense. In short, visual preference is a proxy of attention but does not conclusively measure it. Nonetheless, infants with little or no experience exhibit reliable visual preferences, and some brain processes related to attention *must* subserve them.

To begin this article, we reflected that even newborns and certainly young infants spontaneously attend differently to different sources of stimulation in their environment, an activity that has been observed since the beginnings of developmental science [87], and we asked the question, *Why*? The cascade model which we invoked to account for this observation relates the visual attention-getting power of a stimulus to the level of excitation of stimulus-sensitive neural tissue along the visual pathway in the brain. The present findings do not speak to the deep localization of surface cortical activity, but discriminative ESRP responses aligned with our second hypothesis were clearly measurable at the cortical scalp surface. This taxic mechanism and its utility and functions have potential significance for the early development of sensation, perception, and cognition. For example, these findings document sensory discrimination of saturation by the infant visual system, and they advance understanding of the perceptual organization of infant behavior if only because the early development of object identification and recognition depends wholly on the preverbal infant’s encoding and retention of physical properties of objects, including their color [16,19,35,88–91]. Infants look longer at typically than at atypically colored objects, suggesting early object color knowledge [90]. Color also enters into infant cognition and memory, as infants represent color in visual short-term memory [92], color enhances children’s performance on memory tasks [93], and color is related to recall accuracy in problem solving [94]. Focused attention in infancy predicts standardized cognitive assessments at 2, 3, and 4 to 5 years of age [95], executive functioning at 3 [96] and 5 [97] years of age, as well as experimental tasks and cognitive and meta-cognitive abilities during childhood and adolescence [62]. Attention skills have a foundational role in infants’ information acquisition and learning [98]. Chromatic saturation may also exert a compelling influence on shaping the aesthetics of color preferences. The human visual system discriminates millions of colors [99], but humans appear to express reliable preferences for some colors over others. Color preference is often thought to reflect fluctuating fad, fancy, and framework. However, two key aspects of color preferences have been found to be relatively robust. One is that relatively more saturated colors are preferred over relatively less saturated colors of a fixed dominant wavelength independent of brightness variation, data collection method, developmental age, cultural diversity, and diachronic evaluation [31,32,100–103]. The second (and related) relatively robust color preference is for short (“blue”) and long (“red”) spectral wavelengths relative to mid- spectral (“green” and “yellow”) wavelengths. For example, mean looking times for infants (in paired-comparison and single-stimulus presentation conditions alike) and pleasantness ratings for adults plotted as a function of wavelength parallel one another along the spectrum and converge to show that babies visually prefer and adults rate as more pleasing more naturally saturated colors at the visible spectral extremes—short (blue) and long (red) wavelengths [24,25,28,30,31,35,68,104–106]. Although not all research conforms, and not all cultures have been tested [107], much research since at least 1897 [108] has attested to systematic and reliable adult color preferences across many cultures for blue hues over yellow-green hues [109]. Critically, the infant maxima and minima in preferences are robust to presentation mode (e.g., computer monitor displays as here; Munsell chips in [2,105]; and monochromatic lights in [100]), and these spontaneous preference variations remain after stimuli are equated for brightness [31] and hold across infant gender [30,100]. Together, these consistencies strongly suggest some shared sensory and neurological bases of color preference. One possibility is that these preferences have a similar sensory component in fundamental neural dimensions that underlie early color encoding [110]. Another is that chromatic saturation is implicated in both. Discrimination of saturation may not perfectly predict perceived saturation [111,112], and arguments have been marshalled that purity alone does not account for color preferences [30], but the natural coding of saturation by the nervous system offers a possible basis for both regularities observed in color preferences. Stimuli matched in colorimetric purity still vary in saturation [113–115]. The saturation- discrimination function shows that the blue and red (short- and long- wavelength) ends of the visible spectrum appear naturally as more saturated than green and yellow (middle-wave) portion of the visible spectrum, which is psychologically closer to white. Infant looking times and adult hue pleasantness ratings both parallel the saturation-discrimination function. Evolutionary psychology argues that certain visual stimuli are preferred and that those stimuli possess features which the human visual system likely processes in an optimal fashion. It may be as well that such preferences are to some extent innate or early maturing. Indeed, the color vision of infants as young as 4 months of age aligns with the distribution of colors in natural scenes to which they are exposed [50]. Gibson [116] characterized some infant visual behavior as "captive"–a formulation of behavior that invokes an ecological interpretation. Indeed, many animals exhibit innate taxes to specific stimuli that suggest that specific stimulus-behavior reactions likely serve some vital functions early in life. The spectral transmittance of the oil droplet which covers the laughing-gull chick’s eye corresponds to the spectral reflectance of the red spot on the adult laughing-gull’s mandible. Behavioral color preferences of the laughing-gull’s chick, as manifest in their pecking, follow the same spectral function [117]. Thus, sustenance is assured. Compatible with the present study, Saito [118] proposed a general three-layered structure of color preference based “feelings of ‘pleasantness’” with preferred feelings rooted in brain (amygdalar and hypothalamic) sensitivity forming the nucleus or the innermost first layer, and preferences based on other (individual and environmental) factors composing the surrounding second and third layers. He further speculated that “The closer the preference is to the center of this structure, the more stable it is, and the more it is a preference that is common to all people” (p. 6). As Saito (p. 4) admonished, if such a regular “tendency toward certain [preferences is found], … then … studies should be carried out to investigate whether the cause of such common tendencies in human response is related to an innate cognition style,” as we have done here.

## Conclusions

Attention initiates a cascade for perception, cognition, memory, and action, and specific relations between specific features of the visual environment and their specific processing along the visual system may underlie specific aspects of visual behavior. Here we showed that infants (and adults) selectively look at chromatic stimuli in consonance with the relative saturation of those stimuli and infants’ looking behavior (and adults’ hedonic preference) is likely mediated by central nervous system activity which that saturation excites.
